# Integrated Sample to Detection of Carbapenem-Resistant Bacteria Extracted from Water Samples Using a Portable Gold Nanoparticle-Based Biosensor

**DOI:** 10.3390/s25175293

**Published:** 2025-08-26

**Authors:** Kaily Kao, Evangelyn C. Alocilja

**Affiliations:** 1 Department of Biosystems and Agricultural Engineering, Michigan State University, East Lansing, MI 48824, USA; kaokaily@msu.edu; 2 Global Alliance for Rapid Diagnostics (GARD), Michigan State University, East Lansing, MI 48824, USA

**Keywords:** biosensor, optical biosensing, diagnostics, antimicrobial resistance, carbapenem resistance, genes, pathogens

## Abstract

Antimicrobial resistance (AMR) is a significant global threat and is driven by the overuse of antibiotics in both clinical and agricultural settings. This issue is further complicated by the lack of rapid surveillance tools to detect resistant bacteria in clinical, environmental, and food systems. Of particular concern is the rise in resistance to carbapenems, a critical class of beta-lactam antibiotics. Rapid detection methods are necessary for prevention and surveillance effort. This study utilized a gold nanoparticle-based plasmonic biosensor to detect three CR genes: *bla*_KPC-3_, *bla*_NDM-1_, and *bla*_OXA-1_. Optical signals were analyzed using both a spectrophotometer and a smartphone app that quantified visual color changes using RGB values. This app, combined with a simple boiling method for DNA extraction and a portable thermal cycler, was used to evaluate the biosensor’s potential for POC use. Advantages of the portable bacterial detection device include real time monitoring for immediate decision-making in critical situations, field and on-site testing in resource-limited settings without needing to transport samples to a centralized lab, minimal training required, automatic data analysis, storage and sharing, and reduced operational cost. Bacteria were inoculated into sterile water, river water, and turkey rinse water samples to determine the biosensor’s success in detecting target genes from sample matrices. Magnetic nanoparticles were used to capture and concentrate bacteria to avoid time-consuming cultivation and separation steps. The biosensor successfully detected the target CR genes in all tested samples using three gene-specific DNA probes. Target genes were detected with a limit of detection of 2.5 ng/L or less, corresponding to ~10^3^ CFU/mL of bacteria.

## 1. Introduction

Although AMR is a global concern, low-resource settings are significantly impacted by the contributing factors of AMR and its effects [1,2]. Increased levels of AMR in developing countries can be attributed to the frequent misuse of antibiotics and their unregulated distribution, the use of antibiotics for food animals and crops, and inadequate surveillance and antibiotic susceptibility testing (AST) [3]. Low-resource areas face many challenges regarding antimicrobial resistance, including the lack of healthcare infrastructure and limited access to resources and supplies [4,5]. A weak laboratory or healthcare infrastructure includes inadequate construction for laboratory and waste facilities, low water quality, issues with climate control and ventilation, a lack of disinfectants, and limited internet coverage [6]. There has been progress in closing the gap between AMR surveillance in high- and low-resource countries, in accordance with the World Health Organization (WHO) global action plan [7,8]. However, the surveillance in low-resource areas still needs improvement [9,10]. Another key issue is that traditional phenotypic detection and diagnosis of AMR infections can take several days to complete, and rapid, portable, point-of-care (POC)-based detection methods are crucial for the fight against the spread of AMR [8,11]. One method to improve surveillance efforts is the development of portable, inexpensive (POC) AST methods for gathering data [12,13]. One study created a portable “Mini-Lab” that contained all the necessary equipment, supplies, training, and data management systems needed, and it was used in low-resource hospitals for disease diagnosis and AMR testing in bloodstream infections [14]. One study developed a portable AST method, utilizing a smartphone, to evaluate minimum inhibitory concentrations (MIC) of bacteria that did not require a trained professional to operate [15]. Another study utilized machine learning in an offline smartphone application to analyze disk diffusion test results, which supports its data reporting to improve AMR surveillance initiatives [16]. Another study developed a low-cost, rapid AI-assisted portable system to detect beta-lactam-resistant bacteria in complex environments [17].

Carbapenems, a subclass of beta-lactam antibiotics, are broad-spectrum antibacterials, and their resistance causes high mortality rates in patients with infections [18,19]. Hospital wastewater is a significant source for the spread of carbapenem-resistant (CR) bacteria and CR genes, especially in middle- to low-income countries (LMIC) [20]. This is likely due to the higher levels of carbapenem consumption in LMIC than in high-income countries (HIC) [20]. The groups of carbapenem-resistant *Enterobacterales* (CRE), carbapenem-resistant *Acinetobacter baumanii* (CRAB), and carbapenem-resistant *Pseudomonas aeruginosa* (CRPA) are classified as urgent public health threats by the World Health Organization, and these groups are where the development of new antibiotics is necessary [21]. One method of carbapenem resistance in bacteria is through the production of enzymes, called carbapenemases, that inactivate carbapenem antibiotics [22]. These enzymes are encoded by genes, which are commonly present on mobile elements, like plasmids, of bacterial genomes [23]. Mobile elements promote intercellular exchanges of resistance genes, which are a major contributor to the spread of antibiotic resistance in bacteria [24]. Beta-lactam-resistant genes are separated into classes A, B, and D [25]. Class A (serine penicillinases) includes KPC-producing genes, class B (metallo-beta-lactamases) includes NDM-, IMP-, and VIM-producing genes, and class D (oxacillinases) includes OXA-producing genes [25,26]. Most carbapenem-resistance genes, including *bla*_KPC_, *bla*_NDM-1_, and *bla*_OXA-1_, are located on plasmids [27,28,29,30].

The high cost and long processing time for AMR testing are barriers to global AMR stewardship policy implementation [31]. Traditional methods of AMR gene detection, like conventional PCR and quantitative PCR (qPCR), require only very low levels of DNA and can detect resistance genes in non-cultivable organisms and complex samples [32]. However, PCR-based methods are expensive, require re-optimization for every target gene, and successful detection depends on many factors, including cycle conditions, temperature, primer concentration, and DNA quality and extraction procedures [33,34]. These limitations can make portability challenging. Biosensing technologies provide unique advantages in terms of portability, rapid results, and inexpensive materials [35,36]. One study developed a portable biosensing system utilizing CRISPR/Cas12a and loop-mediated isothermal amplification techniques to detect antimicrobial resistance genes in wastewater from low-resource areas [37]. Another study developed a portable and rapid biosensor to analyze the metabolic activity of bacteria in response to an antibiotic for rapid AST [38]. Rapid POC-based detection of AMR can be achieved using nanomaterials and optical biosensing technologies [39,40]. The development of rapid and accessible biosensing technologies can improve AMR testing methods and surveillance efforts, especially in low-income areas. Biosensing technologies for detecting resistance genes have been mostly validated in clinical samples, but there are few studies focused on water samples [41]. Water plays an important role in AMR transmission, because there are high levels of resistance genes and resistant bacteria in different types of water reservoirs, including sewage and industrial, and pharmaceutical and agricultural wastewater [42]. In addition, many biosensing applications struggle with validation in complex matrices due to sample preparation steps [43].

Plasmonic biosensors often utilize the multifunctional characteristics of gold nanoparticles (GNPs) [44]. GNPs have surface plasmon resonance (SPR) properties that are influenced by their size and shape, and result from the oscillation of free electrons on the surface [45]. These unique properties can be utilized in optical biosensors. This optical biosensor uses dextrin-capped GNPs coated with 11-mercaptoundecanoic acid (MUDA), where the thiol group of the MUDA attaches to the GNP surface and its carboxyl group binds to the amine group of an aminated single-stranded DNA probe. This probe is designed to bind to a specific target sequence. When target DNA is present, the remaining bacterial genome encapsulates the GNPs. This encapsulation prevents GNPs from aggregating when hydrochloric acid (HCl) is added. Small (around 20–30 nm in diameter) and dispersed GNPs have a maximum oscillation near 520 nm and have a red appearance [46]. When GNPs aggregate, the solution changes from red to blue, which is caused by the peak oscillation range shift [47]. This optical biosensor uses this feature of GNPs to differentiate between a target and a non-target based on the level of GNP aggregation. This method can be completed within 45 min after DNA extraction and does not require gene amplification. AI-based analysis, while having tremendous potential for diagnostics, was not used in this study, as the measurement and data analysis processes for the biosensor have not yet been adapted and validated for AI integration. Manual analysis was conducted.

The goal of emerging methods of pathogen detection is to decrease identification time. One challenge for bacterial detection from samples is the time required for sample preparation [48]. To identify bacteria from samples, the bacteria must first be separated from the sample matrix and ideally concentrated to avoid lengthy sample enrichment steps [49]. There are many methods of bacterial separation and concentration, including chemical, physical, physio-chemical, and biological methods [49]. Magnetic nanoparticles (MNPs) have gained interest in procedures where bacterial concentration is necessary, due to their functionalization capabilities and ability to capture and concentrate bacterial cells [50]. MNPs attach to bacterial cells in multiple ways based on surface functionalization, including antigen–antibody binding, carbohydrate–lectin interactions, electrostatic interactions, and covalent binding [51]. Glycan (chitosan)-coated MNPs have been successful for bacterial capture in several studies [43,48,52,53,54].

This study aimed to create a proof-of-concept multi-probe, portable, and rapid biosensing method for carbapenem-resistant bacteria in water samples using glycan-coated MNPs to capture and concentrate bacteria.

## 2. Materials and Methods

### 2.1. Materials

Frozen bacterial stock cultures of *E. cloacae* and *S. aureus* were obtained from the Nano-Biosensors Laboratory at Michigan State University (MSU). Susceptible *E. coli* C-3000 (15597) and resistant *E. coli* BAA-2471 and *E. coli* BAA-2340 were obtained from the American Type Culture Collection (ATCC). A NanoDrop One spectrophotometer (Thermo Fisher Scientific, Waltham, MA, USA) was used to measure absorbance spectra. A Qubit Fluorometric Quantification device (Thermo Fisher Scientific, Waltham, MA, USA) was used to quantify DNA concentration. A portable thermal cycler (VWR International, Radnor, PA, USA) was used in place of a standard laboratory-size thermal cycler. A Zetasizer (Malvern Panalytical, Westborough, MA, USA) was used to measure the zeta potential of bacteria and magnetic nanoparticles suspended in water. Oligonucleotide probes and PCR primers were ordered from Integrated DNA Technologies (IDT). Tryptic Soy Agar (TSA), Tryptic Soy Broth (TSB), hydrochloric acid, gold (III) chloride, sodium carbonate, 11-mercaptoundecanoic acid, phosphate-buffered saline (PBS), citric acid, sodium dodecyl sulfate, CHROMagar *Staph aureus*, CHROMagar *E. coli*, MacConkey agar, and dextrin were purchased from Sigma Aldrich (St. Louis, MO, USA). Glycan-coated MNPs were synthesized in the Nano-Biosensors Lab at MSU [54].

### 2.2. Bacterial Cultures

The frozen stock cultures of bacteria were stored at negative 80 °C. Bacteria stock cultures were streaked on plates and incubated at 37 °C for 24 h to create master plates. *E. coli* species were plated on *E. coli* selective agar, *S. aureus* was plated on *S. aureus* selective agar, and *E. cloacae* was plated on MacConkey agar. Broth cultures were created by transferring one bacterial colony into 9 mL of TSB and grown overnight.

### 2.3. Oligonucleotide Probe Design and PCR Verification

CR genes *bla*_NDM-1_, *bla*_OXA-1_, and *bla*_KPC-3_ were detected using oligonucleotide probes. The probe sequences were designed utilizing the National Center for Biotechnology Information Basic Location Alignment Search Tool (NCBI BLAST). NCBI BLAST (https://blast.ncbi.nlm.nih.gov/Blast.cgi accessed on 12 August 2025) confirmed that the probe sequence was specific to organisms containing the respective target genes. The probe sequences are shown in Table 1.

PCR, followed by gel electrophoresis, was used to confirm the presence or absence of resistance genes in bacteria from pure cultures and captured cells. Forward and reverse primers and thermal cycling conditions, from previous studies, were used for target gene detection. It was confirmed that the primers were located within the gene of interest. Each reaction included 12.5 μL of PCR Master Mix reagent (Qiagen, Germantown, MD, USA), 2.5 μL of 2 μM F-primer, 2.5 μL of 2 μM R-primer, 6.5 μL of water, and 1 μL of DNA template for a total reaction volume of 25 μL. After thermal cycling, the reactions were stained with 6x loading dye and Sybr and run on a 2.0% agarose gel. PCR methods, including primers and amplification steps, were used solely for validation of gene presence. The biosensor assay did not require amplification for DNA and oligo probe interactions.

A set of forward and reverse primers were used for *bla*_NDM-1_ detection, from a previous study [55]. The thermal cycling conditions included an initial heating step at 95 °C for 5 min, followed by 35 cycles of 94 °C for 30 s, 55 °C for 30 s, and 72 °C for 30 s, and extension at 72 °C for 10 min [55]. The primer sequences are shown in Table 1. The estimated amplicon length was 509 bp.

The PCR detection of the *bla*_OXA-1_ gene utilized primers and thermal cycling conditions from a previous study [56]. The thermal cycling conditions included initial heating at 94 °C for 5 min, followed by 32 cycles of 94 °C for 30 s, 52 °C for 30 s, and 72 °C for 60 s, and extension at 72 °C for 10 min [56]. The primer sequences are shown in Table 1. The estimated amplicon length was 619 bp.

The PCR detection of the *bla*_KPC-3_ gene utilized primers and thermal cycling conditions from a previous study [57]. The thermal cycling conditions included initial heating at 94 °C for 10 min, followed by 36 cycles of 94 °C for 30 s, 52 °C for 40 s, and 72 °C for 50 s, and extension at 72 °C for 5 min [57]. The primer sequences are shown in Table 1. The estimated amplicon length was 574 bp.

**Table 1:** Single-stranded DNA probe and PCR forward and reverse primers sequences, length or amplicon length, and melting temperature (Tm).

CR Gene	Probe or Primer Sequence (5′ to 3′)	Amplicon Length (bp)	Tm (°C)	Source
*bla*_NDM-1_	Probe: CAACACAGCCTGACTTTCGCCGCCAATGGCTGGGTCGAACCAGCAACCGC	50	74.4	[58]
	F-primer: CAGCACACTTCCTATCTC	18	49.5	[55]
	R-primer: GTAGTGCTCAGTGTCGGCAT	20	57.2	[55]
*bla*_OXA-1_	Probe: CGATGCATCCACAAACGCTGAAATTGCTCAATTCAATAAAGCAAAGTGTG	50	66.2	[58]
	F-primer: ATATCTCTACTGTTGCATCTCC	22	59.3	[56]
	R-primer: AAACCCTTCAAACCATCC	18	57.5	[56]
*bla*_KPC-3_	Probe: CGGTGTGTACGCGATGGATACCGGCTCAGGCGCAACTGTAAGTTACCGCGCTGAGGA GCG	60	74.0	This study
	F-primer: CGGTGTGTACGCGATGGATA	20	56.9	[57]
	R-primer: TCCGGTTTTGTCTCCGACTG	20	57.1	[57]

### 2.4. DNA Extraction Method

To explore using a low-cost, portable methodology, a simple boiling procedure was used to extract bacterial DNA. First, 1.5 mL of an overnight culture of bacteria was centrifuged at 8000 rpm for 3 min. Then, the supernatant was removed, and the cell pellet was resuspended in elution buffer 1x AE. Next, the tubes were boiled at 99 °C for 20 min. Then, the tubes were put inside the freezer at −20 °C for 5 min. Finally, the tubes were centrifuged at 15,000 rpm for 2 min, and the top half of the supernatant was used for the assay. The DNA quality was quantified using the A_260_/A_280_ ratio measured with a Nanodrop One (Thermo Fisher Scientific). All DNA extracts used in this study had a A260/A280 between 1.80 and 2.0 to ensure high quality. The DNA concentration was quantified using a Qubit Fluorometric Quantification device (Thermo Fisher Scientific).

### 2.5. Biosensor Design and Principle

The biosensor uses oligonucleotide probes, GNPs, and bacterial DNA. In each tube, 5 μL of probe, 5 μL of GNPs, and 10 μL of DNA were combined. The probes were diluted to a concentration of 25 μM using 1x TE elution buffer. Tubes were heated in a portable thermal cycler to allow probe hybridization. The thermal cycling conditions included 1 cycle of 5 min at 95 °C to denature the sample DNA, 10 min at 55 °C to anneal the DNA to the probe, and 1 min at 27 °C to cool. This cycle allows target DNA to hybridize to the probe, if it is present in the sample.

**Figure 1:**
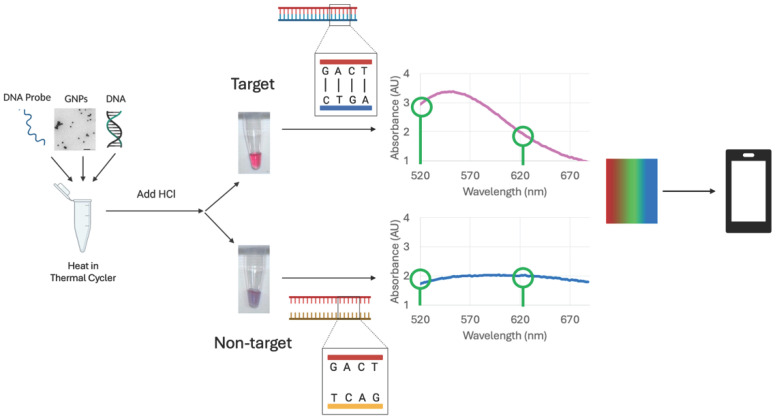
Optical biosensor assay utilizing unique SPR properties of GNPs to distinguish between samples containing bacteria with CR genes (positive) and samples not containing the target genes (negative). The aminated oligonucleotide probe binds to the carboxyl group of the MUDA on the GNP surface, and, if target DNA is present, the probe binds to the target gene sequence, causing the sample to remain red. If the target sequence is not present, the sample turns blue. At 520 nm, red light is reflected, and green light is absorbed. At 620 nm, blue light is reflected, and orange light is absorbed. Therefore, the color change can be quantified using the spectral ratio of A_520_/A_620_ to indicate how red or blue the sample is. This can also be quantified using an RGB cellphone app. (created with https://www.biorender.com/ accessed on 30 June 2025).

The biosensor leverages the surface plasmon resonance (SPR) properties of GNPs that arise from the collective oscillation of free electrons on the surface [59]. This oscillation peaks at a specific frequency or SPR, and results in an absorption of light that can be measured using a spectrophotometer [59]. The SPR peak location depends on the size of the GNPs [59,60]. It was shown that GNPs smaller than 30 nm have a maximum oscillation range of 500 nm to 540 nm [60,61]. The GNPs used in this assay range from 16.61 to 22.53 nm in diameter, with a peak absorption wavelength between 518 and 522 nm. When GNPs agglomerate, an SPR band shifts, producing a visible color change [60]. This phenomenon was used to differentiate target (positive) from non-target (negative) samples by adding HCl to induce aggregation. The MUDA-coated, dextrin-capped GNPs link with the aminated probe to create a GNP–probe complex. When target DNA binds to the probe, the bacterial genome surrounds the GNPs, preventing aggregation and signaling a positive result. In contrast, aggregation occurs in the absence of target DNA, indicating a negative sample. The distance-dependent nature of the SPR band enables quantification of this color change using a spectrophotometer or a cellphone app. Figure 1 shows the principle behind the biosensor assay utilizing the SPR properties of GNPs to distinguish between samples that do and do not contain target genes.

The added volume of 0.1 M HCl was optimized for the *bla*_KPC-3_ probe. Optimization testing was conducted in a previous study for the *bla*_NDM-1_ and *bla*_OXA-1_ probes [58]. The optimal volume was determined by adding increasing volumes of HCl, from 4 μL to 8 μL in 1 μL increments, to separate trials containing target, non-target, and control samples. Target and non-target DNA were standardized to 2.5 ng/μL. The optimal volume occurred when the target sample remained visibly red, while the non-target sample and control turned blue after 10 min.

### 2.6. GNP Synthesis

**Figure 2:**
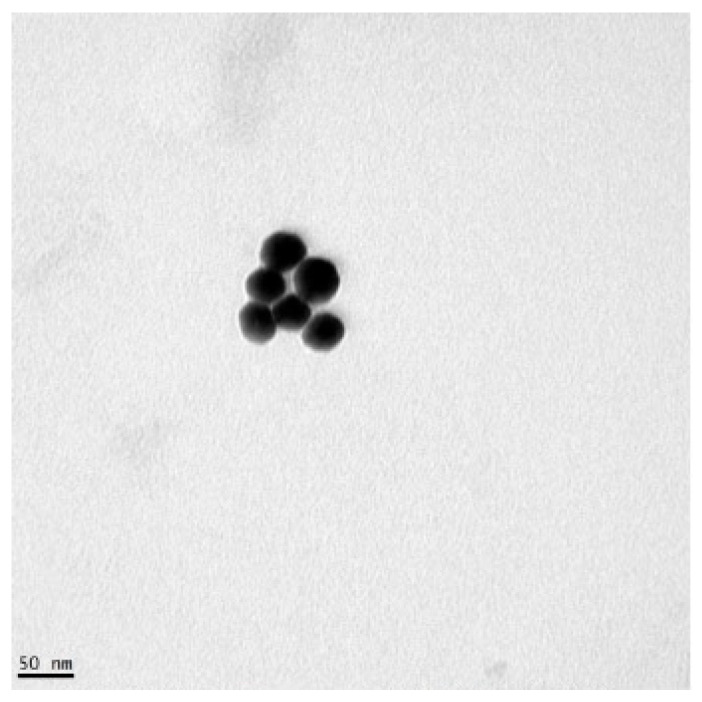
TEM image of GNPs.

Gold nanoparticles (GNPs) were synthesized according to a method previously described [62]. The dextrin-capped GNP synthesis used gold (III) chloride trihydrate (HAuCl_4_), sodium carbonate (Na_2_CO_3_), dextrin, and sterile water. The GNPs were thiol-coated using 11-mercaptoundecanoic acid (MUDA) and suspended in borate buffer. The GNPs were stored at 4 °C before use. The GNPs used in this study had a peak absorbance wavelength between 518 and 522 nm. To characterize the size of the GNPs, TEM images were taken as part of a previous study, and a sample of 40 GNP diameters were measured [60]. The average diameter and standard deviation was 19.64 ± 1.39 nm and all diameters ranged from 16.61 to 22.53 nm [58]. A TEM image of GNPs synthesized using the same method as those used in this study is shown in Figure 2.

### 2.7. Limit of Detection in Pure Cultures

A series of 5 trials for the *bla*_KPC-3_ probe was conducted to determine the biosensor’s limit of detection (LOD) when using DNA extracted from pure cultured bacteria. LOD testing for *bla*_NDM-1_ and *bla*_OXA-1_ was conducted in a previous study [58]. All the bacterial DNA was extracted using the boiling method. The DNA was diluted to concentrations ranging from 2.5 to 0.15625 ng/μL. For each trial, the sample containing the target DNA was compared to a non-target sample (*E. coli* C-3000) of the same concentration and a control (nuclease-free water). The target (*E. coli* BAA-2340) and non-target bacteria were obtained from ATCC. The optimized volume of HCl was added, and the absorbance was measured with the Nanodrop One after ten minutes. The A_520_/A_620_ ratios for the target, non-target, and control samples were compared to determine when the target sample was no longer distinguishable from the non-target at the same concentration, or the control, based on the 95% confidence intervals.

### 2.8. Magnetic Nanoparticles for Bacterial Concentration

**Figure 3:**
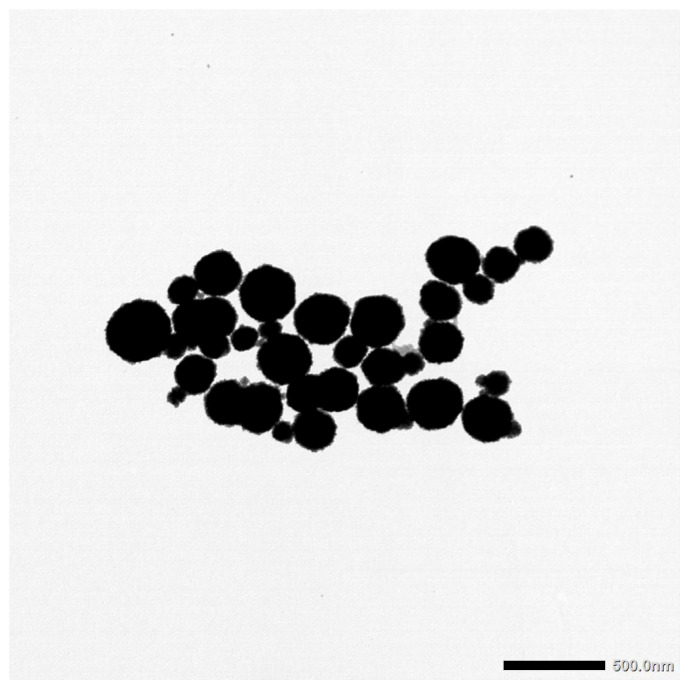
TEM image of MNPs.

To determine whether the biosensor could detect target DNA extracted from water sample matrices, magnetic nanoparticles were utilized for bacterial capture and concentration from inoculated type 1 water, river water, and turkey processing rinse water samples. For sample inoculation, 4 h spiked bacterial cultures were serially diluted to 10^−3^ to estimate a total inoculation level of ~10^5^ CFU/mL. This was confirmed by plating after each experiment. After dilution, 1 mL of culture was added to 25 mL of each water sample in a 50 mL conical tube. Then, the sample was diluted with 15 mL of PBS, with an adjusted pH of 4. The pH of the PBS was adjusted using 1 M HCl to improve the capture effects, which was shown in previous work [43,63]. After mixing, 1 mL was removed for a control (before MNP addition). This control was plated on selective media in triplicate to determine the number of bacteria in the sample before MNP concentration. Then, 0.4 mL of MNPs were added to the tube and incubated at room temperature for 10 min. Figure 3 shows a TEM image of the MNPs used in this study.

**Figure 4:**
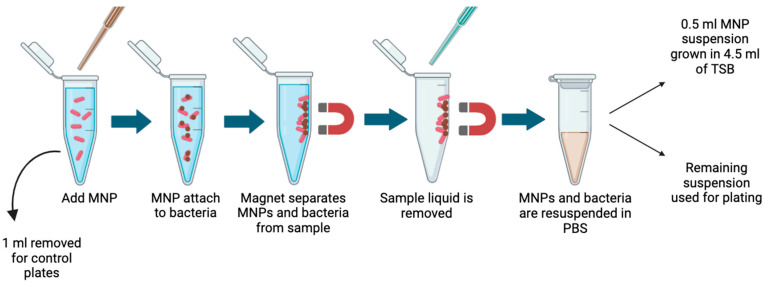
MNP capture procedure for bacteria in water (created with https://www.biorender.com/ accessed on 30 June 2025).

The MNPs attach to the bacteria due to electrostatic attraction coupled with the glycan–protein interaction between the MNP and bacterial proteins [43,63]. The tube was then placed on a magnetic rack at room temperature for 5 min to allow magnetic separation. Then, the supernatant was removed, and the remaining MNPs were resuspended in 1 mL of PBS. This procedure was performed in triplicate for each bacterium. This process is shown in Figure 4.

After resuspension, 500 μL of the MNP resuspension was added to 4.5 mL of TSB and grown for 6 h. Then, the DNA extraction procedure using a boiling method, described above, was performed. The MNP resuspension was also plated on selective media in triplicate to obtain colony counts indicating the number of cells captured by the MNP. The colony counts from the control plates (before MNPs) and the MNP plates (captured cells) were compared to determine the concentration factor and capture efficiency. Equation (1) describes the concentration factor based on the plate counts from before and after MNP extraction. Equation (2) describes the capture efficiency based on plate counts before and after MNP extraction and total sample volumes.

Equation (1): Concentration Factor

(1) $$CF=\frac{CFU\;in\;MNP\;treated\;sample}{CFU\;in\;non\;treated\;control}$$

Equation (2): Capture Efficiency

(2) $$CE=\frac{\log\left(\frac{CFU}{mL}\right)\;before\;MNP\;capture}{\log\left(\frac{CFU}{mL}\right)\;after\;MNP\;capture}=\frac{\log\left(\frac{CFU\;before}{10\;\mu L}*\left(\frac{1000}{1\;mL}\right)\right)}{\log\left(\frac{CFU\;after}{10\mu L}*\left(\frac{1000}{1\;mL}\right)*\left(\frac{1\;mL}{40\;mL}\right)\right)}$$

**Figure 5:**
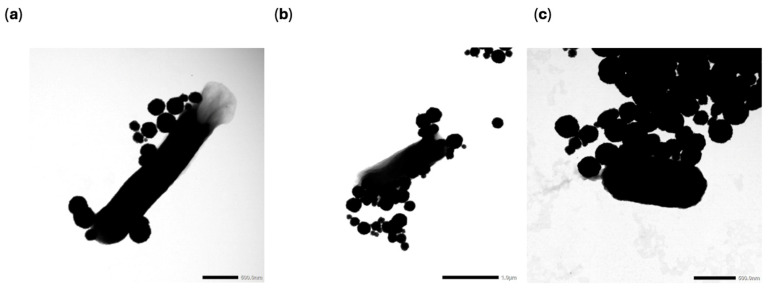
(**a**) TEM images of resistant *E. coli* BAA-2340, (**b**) TEM image of resistant *E. coli* BAA-2471, and (**c**) TEM image of susceptible *E. coli* C-3000.

To visualize the MNP-bacterial binding of the *E. coli* species, TEM imaging was performed. A 0.1% uranyl acetate stain was used after the samples were fixed using a 2.5% glutaraldehyde solution and 0.1 M cacodylate buffer wash. Images were taken in the range of 5000–25,000× magnification. All three bacterial species were observed to have interactions with multiple MNPs, and no significant differences in these interactions were seen between species. This is shown in Figure 5.

### 2.9. Zeta Potential for MNP and Bacterial Characterization

To analyze surface properties of *E. coli* and MNPs, a Zetasizer (Malvern) was used to measure the zeta potential of the surfaces. The zeta potential is an indicator of the electrostatic charge of particles [64]. One mL of an overnight bacterial transfer was centrifuged at 8000 rpm for 3 min and the cell pellet was resuspended in 1 mL of sterile water, diluted 1:10 in sterile water, and placed in the cuvette. One mL of an MNP suspension in sterile water (50 mg MNP/10 mL water) was diluted 1:10 in sterile water and placed in the cuvette. The Zetasizer (Malvern) zeta potential measurement was taken for three trials for each test organism and the MNPs.

### 2.10. Multi-Probe, Portable Detection for MNP-Captured Cells

**Figure 6:**
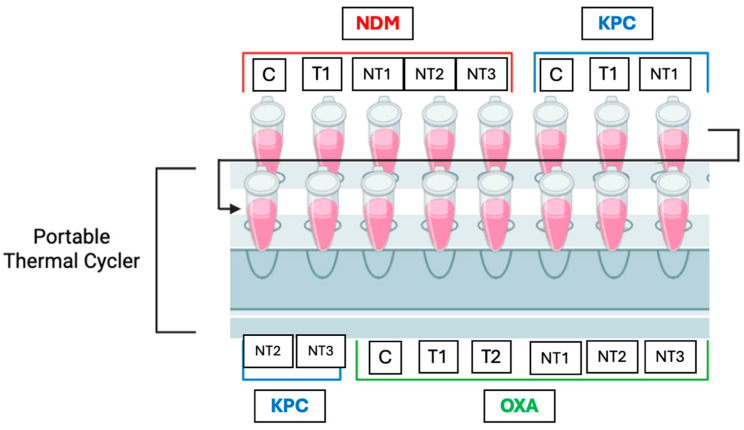
Portable thermal cycling set up to include one trial for each probe in a standardized tube placement order.

Three trials were conducted for each probe using the DNA extracted from inoculated water samples. Type 1 water samples were inoculated with ~10^5^ CFU/mL and extracted using MNPs in the abovementioned methods. For each trial, a control, target(s), and three non-targets were tested for all probes. The target for *bla*_NDM-1_ was *E. coli* BAA-2471 and the target for *bla*_KPC-3_ was *E. coli* BAA-2340. For the *bla*_OXA-1_ probe, both *E. coli* BAA-2471 and *E. coli* BAA-2340 were used as the targets. For all probes, *E. coli* C-3000 (NT1), *E. cloacae* (NT2), and *S. aureus* (NT3) were used as non-targets for comparison. The genes present in the inoculated samples were confirmed using the PCR methods described above. To explore the biosensor’s portability, a hand-held, portable thermal cycler (VWR International) was used instead of a standard-sized laboratory thermal cycler. For each thermal cycling procedure, one trial from each probe was included in the thermal cycle. The thermal cycler had a total of 16 tube positions, and one run accommodated one trial for each probe (5 tubes for *bla*_NDM-1_, 5 tubes for *bla*_KPC-3_, and 6 tubes for *bla*_OXA-1_). The order of tube placement was standardized to provide a simple method of loading and unloading the thermal cycler, while keeping track of which tubes contained which probe. This placement is shown in Figure 6.

In addition to the portable thermal cycler, an RGB-measuring cellphone application was used to analyze images of the sample tubes. A picture of the biosensor’s colorimetric output is taken and fed into the biosensor’s RGB app, an image processing algorithm. The RGB model translates the visual components: Pure red = (255, 0, 0); Pure green = (255, 0, 0); Pure blue = (0, 0, 255); White = (255, 255, 255); and Black = (0, 0, 0). As the app processes the image, each pixel stores RGB values and displays them on the smartphone screen. To ensure consistency and reduce the effects of environmental factors, such as picture lining, RGB values are normalized against the control tube in each image. This control serves as a reference point for signal-to-noise standardization. This ratio correlates to the A520/A620 absorbance values obtained from the spectral data.

The A_520_/A_620_ ratio from spectral measurements was used to compare targets and non-targets to the control tube. If the sample had a statistically higher response than the control, based on 95% confidence intervals, it was classified as a positive response. If the sample was not statistically higher than the control, based on 95% confidence intervals, it was classified as a negative response.

## 3. Results

### 3.1. Optimization and Limit of Detection in Pure Cultures

**Figure 7:**
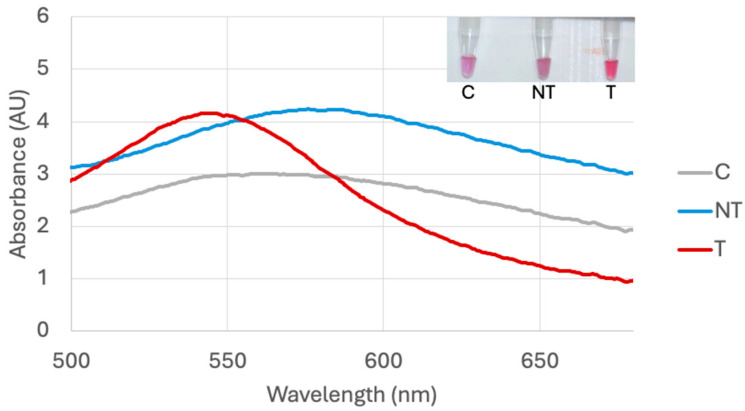
*bla*_KPC-3_ probe biosensor response 10 min after adding 8 μL 0.1 M HCl for target *E. coli* BAA-2340 (T), non-target *E. coli* C-3000 (NT), and water control (C).

The optimal HCl volume was determined by adding varying volumes of HCl and observing the color change in the biosensor response in a target, non-target, and control sample 10 min after HCl addition. The optimal volume occurred when the target sample remained visibly red and the non-target and control turned blue. Optimization testing for *bla*_NDM-1_ and *bla*_OXA-1_ was conducted in a previous study, and resulted in optimal volumes of 5 μL and 6 μL, respectively, for DNA extracted through boiling [58]. The *bla*_KPC-3_ probe visual and spectral result for the optimal 8 μL are shown in Figure 7.

**Figure 8:**
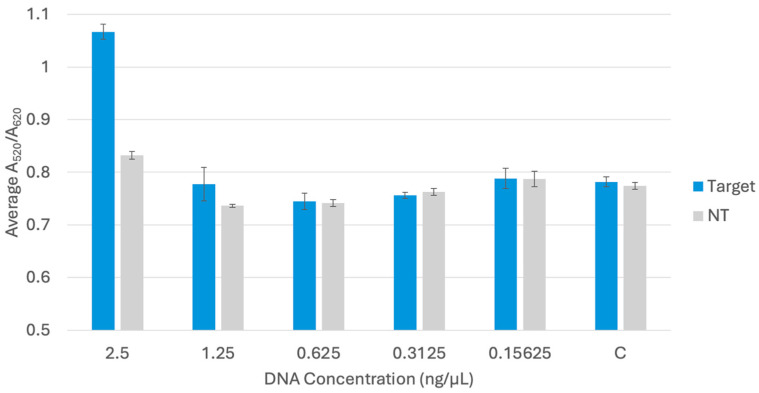
LOD testing for *bla*_KPC-3_ comparing target *E. coli* BAA-2340 (Target), non-target *E. coli* C-3000 (NT), and water control (C).

A limit of detection (LOD) test was conducted to determine the lowest concentration of target DNA extracted from pure cultures that could be detected by the biosensor. LOD testing was conducted for *bla*_NDM-1_ and *bla*_OXA-1_ in a previous study, following the same procedure, and resulted in an LOD of 0.625 ng/μL for both probes with pure culture DNA extracted through boiling [58]. A series of five trials was conducted for the *bla*_KPC-3_ probe, and the average A_520_/A_620_ was compared between target (*E. coli* BAA-2340) and non-target (*E. coli* C-3000) samples at the same concentration and a water control. The LOD results for *bla*_KPC-3_ are shown in Figure 8.

In a series of five trials, comparing the 95% confidence intervals of the target, non-target, and control samples, it was statistically determined that the LOD for the *bla*_KPC-3_ probe in pure cultures was 2.5 ng/μL, since this is the lowest concentration that the confidence interval did not overlap with the confidence interval of control samples.

### 3.2. MNP Bacterial Capture

A series of three trials was conducted for each organism captured from water samples. Plate counts before MNP addition and after MNP addition were compared to calculate the concentration factor (CF), as shown in Equation (1). Capture efficiency was calculated as shown in Equation (2). Table 2 shows the average CF and standard error for the resistant and susceptible bacteria used in this study.

A CF higher than 1.0 indicates increased bacterial capture and shows that the addition of MNPs increases the relative number of cells or CFUs per volume after removing the liquid sample and resuspending the MNPs and bacteria in a smaller volume. As shown in Table 1, each bacterial strain has a CF significantly larger than 1, except for *E. coli* BAA-2471.

### 3.3. Zeta Potential Measurements for E. coli and MNPs

**Figure 9:**
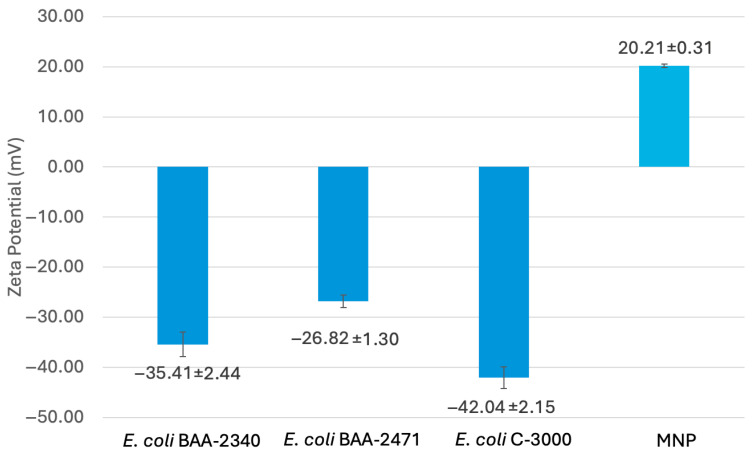
Zeta potential of bacteria and MNPs. *E. coli* 2471 and *E. coli* 2340 are carbapenem-resistant; *E. coli* C-3000 is carbapenem-susceptible.

To estimate the surface charge of *E. coli* and MNPs, a Zetasizer (Malvern) was used to measure the zeta potential of the surfaces in triplicate. Figure 9 shows the results from the zeta potential measurements for the resistant *E. coli* (BAA-2340 and BAA-2471), the susceptible *E. coli* C-3000, and MNPs in water.

*E. coli* BAA-2471 had a less negative zeta potential than *E. coli* BAA-2340 and *E. coli* C-3000 based on the average and 95% confidence intervals. Resistant *E. coli* BAA-2340 and *E. coli* BAA-2471 had less negative zeta potentials compared to the susceptible *E. coli* C-3000. The MNPs had a positive zeta potential. It was shown that positively charged MNPs have an increased bacterial capture when compared to negatively charged MNPs [65]. A positively charged glycan coating on MNPs could reduce electrostatic repulsion between bacterial cells and MNPs [66]. Electrostatic attraction is only one potential mechanism of binding between MNPs and bacteria. As previously mentioned, bacteria–MNP binding occurs due to a combination of glycan–protein interactions and electrostatic effects.

### 3.4. Detection of MNP-Captured Cells

**Figure 10:**
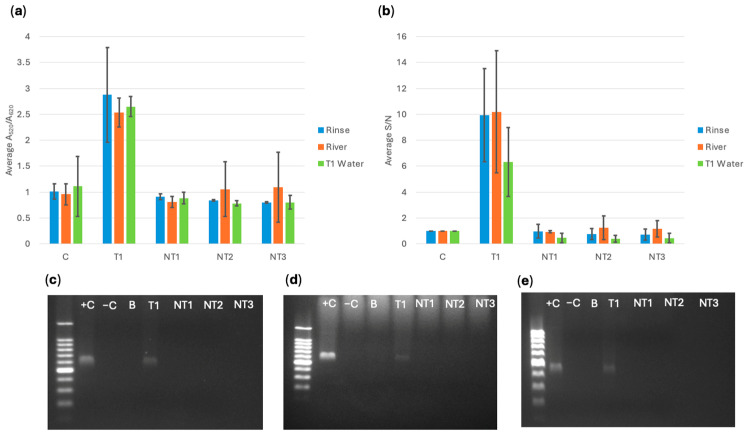
(**a**) *bla*_NDM-1_ probe spectral average A_520_/A_620_ ratio compared to (**b**) cellphone average S/N value comparing *E. coli* BAA-2471 (T1) to *E. coli* C-300 (NT1), *E. cloacae* (NT2), *S. aureus* (NT3) extracted from a water sample and a water control, and PCR verification for *bla*_NDM-1_ in bacteria magnetically captured from (**c**) turkey rinse, (**d**) river water, and (**e**) type 1 water using pure culture *E. coli* BAA-2471 (+C), *E. coli* BAA-2340 (−C), a blank of AE buffer (B), and MNP-extracted bacterial DNA *E. coli* BAA-2471 (T1), *E. coli* C-3000 (NT1), *E. cloacae* (NT2), and *S. aureus* (NT3).

**Figure 11:**
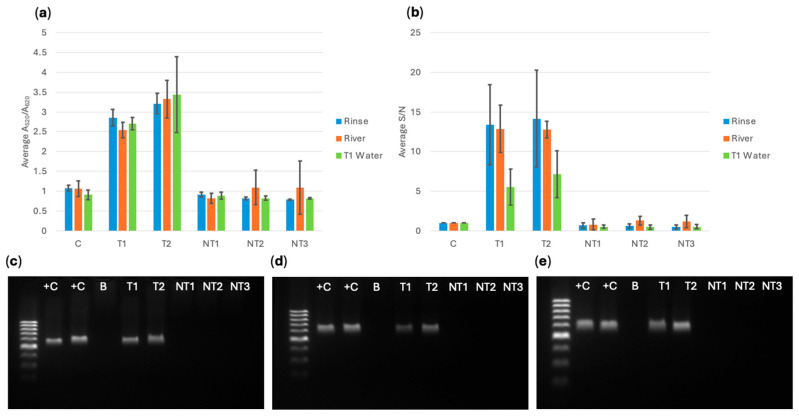
(**a**) *bla*_OXA-1_ probe spectral average A_520_/A_620_ ratio compared to (**b**) cellphone average S/N value comparing *E. coli* BAA-2471 (T1) and *E. coli* BAA-2340 (T2) to *E. coli* C-300 (NT1), *E. cloacae* (NT2), *S. aureus* (NT3) extracted from a water sample and a water control, and PCR verification for *bla*_OXA-1_ in bacteria magnetically captured from (**c**) turkey rinse, (**d**) river water, and (**e**) type 1 water using pure culture *E. coli* BAA-2471 (+C), *E. coli* BAA-2340 (+C), a blank of AE buffer (B), and MNP-extracted bacterial DNA *E. coli* BAA-2471 (T1), *E. coli* BAA-2340 (T2), *E. coli* C-3000 (NT1), *E. cloacae* (NT2), and *S. aureus* (NT3).

**Figure 12:**
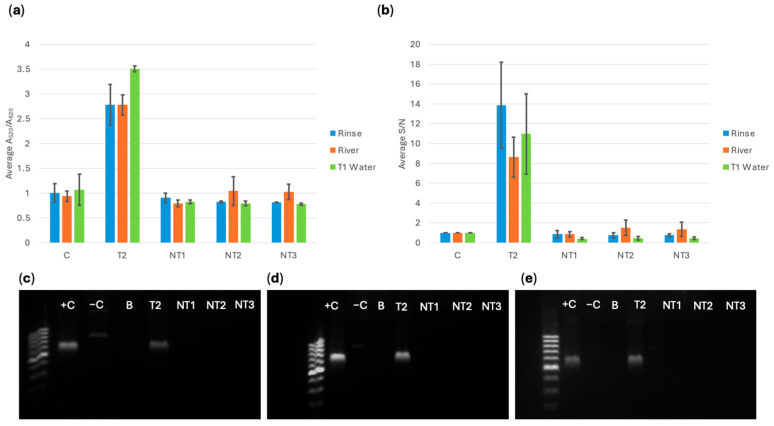
(**a**) *bla*_KPC-3_ probe spectral average A_520_/A_620_ ratio compared to (**b**) cellphone average S/N value comparing *E. coli* BAA-2340 (T2) to *E. coli* C-300 (NT1), *E. cloacae* (NT2), *S. aureus* (NT3) extracted from a water sample and a water control, and PCR verification for *bla*_KPC-3_ in bacteria magnetically captured from (**c**) turkey rinse, (**d**) river water, and (**e**) type 1 water using pure culture *E. coli* BAA-2340 (+C) and *E. coli* BAA-2471 (−C), a blank of AE buffer (B), and MNP-extracted bacterial DNA *E. coli* BAA-2340 (T2), *E. coli* C-3000 (NT1), *E. cloacae* (NT2), and *S. aureus* (NT3).

After the bacteria were captured using MNPs, the concentrated solution was grown for 6 h. Then, DNA extraction was conducted using thermal lysis (boiling). The lowest DNA concentration obtained from the extraction was 6 ng/μL, so all DNA was standardized to this value when conducting the biosensor detection. Figure 10, Figure 11 and Figure 12 show the spectral A_520_/A_620_ ratio compared to the RGB app results for biosensor response with DNA extracted from captured cells, and PCR electrophoresis using pure culture DNA and DNA extracts from MNP captured bacterial cells in turkey rinse, river, and type 1 water samples. A series of three trials (*n* = 3) were performed for each experiment, and 95% confidence intervals were calculated using standard error and t-distribution. If the sample had a statistically higher response than the control, based on confidence intervals, it was classified as a positive response. If the sample did not have a statistically higher response, it was classified as a negative response.

For all three probes, the target gene was successfully detected and differentiated from the control and the susceptible non-targets. The app data showed very similar trends and resulted in identical conclusions compared to the spectral analysis. It was observed that the variability in the app data was more significant compared to the spectral data. External factors like reflections on tube surfaces and differences in placement of photo RGB measurement zone could cause this variation. Even with a low capture for *E. coli* BAA-2471, it could still be detected by the biosensor. PCR results confirmed that *E. coli* BAA-2340 contained *bla*_KPC-3_ and *bla*_OXA-1_ and *E. coli* BAA-2471 contained *bla*_NDM-1_ and *bla*_OXA-1_.

## 4. Discussion

Using a portable, rapid, and inexpensive method for detecting CR genes can help improve the accessibility of current testing methods. A plasmonic DNA-based biosensor was used in this study, and it successfully detected CR genes *bla*_NDM-1_, *bla*_OXA-1_, and *bla*_KPC-3_ using a simple boiling DNA extraction procedure, a portable thermal cycler, and an RGB cellphone app to measure the biosensor response. This study detected CR genes from bacteria-inoculated water samples using magnetic nanoparticles to concentrate the bacteria before DNA extraction. The potential practical application of this technology includes an alternative to POC and AST methods for identifying CR in human, animal, food, and environmental samples without needing time-consuming sample preparation methods or expensive materials.

All bacteria were successfully captured and concentrated by the MNPs, except for *E. coli* BAA-2471. The impact of low CF and CE of *E. coli* BAA-2471 in all matrices impacts field deployment for this biosensing technology. The low capture means that a significant amount of this target bacteria remains unbound to the MNPs and is lost during separation. This means there could be variability in detection performance, which is problematic when unknown strains are present in samples. To combat low concentration factor, incubation time can be increased to ensure enough bacteria are present in the sample for detection. The CF and CE for all bacteria were lower in both turkey rinse and river water samples, which indicates significant matrix effects on successful capture.

**Figure 13:**
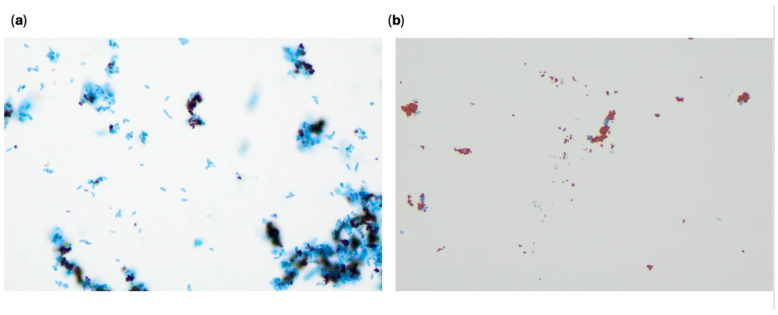
(**a**) *E. coli* BAA-2340 with MNP forming large bacteria–MNP groupings (**b**) *E. coli* BAA-2340 with MNPs showing detailed image of multiple bacteria bound with MNPs.

**Figure 14:**
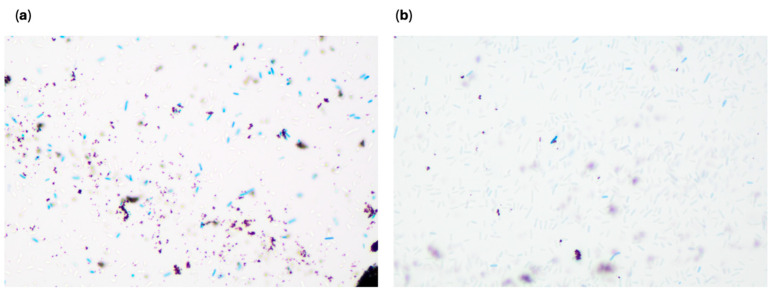
(**a**) *E. coli* BAA-2471 with MNP with limited clumping and MNP–bacterial binding and free MNPs without bacterial binding; (**b**) *E. coli* BAA-2471 showing limited methylene blue-dyed cells and MNP–bacterial binding.

A microscopy visualization was conducted to visualize any differences between this organism and the resistant *E. coli* BAA-2340 with high CF. One mL of a 4 h bacterial spike was centrifuged at 8000 rpm for 3 min. The pellet was resuspended in sterile water. Then, MNPs were added, and the tube was incubated at room temperature for 5 min. Figure 13 and Figure 14 show bacteria–MNP binding and illustrate some differences between the target bacteria *E. coli* BAA-2340 and *E. coli* BAA-2471.

The main differences noted were that the sample with *E. coli* BAA-2471 and MNPs formed fewer large bacteria–MNP groupings than *E. coli* BAA-2340 and appeared to have less attachment between MNPs and the bacteria. The Zetasizer can be used to measure particle size. To analyze particle size, 100 μL of the MNP concentrated solution was mixed with 900 μL of sterile water in a cuvette. Then, the Zetasizer particle size module of the instrument was run for three separate trials with *E. coli* BAA-2340 and *E. coli* BAA-2471. This resulted in an average particle diameter of 1858.67 ± 177.99 nm for *E. coli* BAA-2340 with MNPs and 855 ± 106.15 nm for *E. coli* BAA-2471 with MNPs. This confirms that the *E. coli* BAA-2340 forms larger clumping when combined with MNPs compared to *E. coli* BAA-2471. Another difference noted was that the visualization dye, methylene blue, did not successfully dye all the bacteria shown in the *E. coli* 2471 slide. Methylene blue is a positively charged substance, and binds to the negatively charged lipopolysaccharide (LPS) of Gram-negative bacteria and other negatively charged components like DNA, RNA, and polyphosphates [67,68]. One of the methods of resistance in bacteria is changing surface properties like permeability and binding proteins to reduce binding between antibiotics and bacteria [69]. Each bacterial species has unique surface properties, and it has been shown that the development of antimicrobial resistance can result in cell property changes involving adhesion to host surfaces [43]. This could occur in addition to the production of enzymes used to break down antibiotics, like carbapenemases [70]. Combining the less-negative zeta potential of *E. coli* BAA-2471 and the lack of dyed cells compared to *E. coli* BAA-2340 indicates that the surface properties are different. These differences could explain the lack of MNP capture. It was also observed that the resistant strains of *E. coli* BAA-2340 and BAA-2471 had less negative zeta potentials compared to the susceptible strain of *E. coli* C-3000. The concentration factor for *E. coli* BA-2471 was lower than that of *E. coli* BAA-2340. Correspondingly, the zeta potential of *E. coli* BAA-2471 is less negative than that of *E. coli* BAA-2340. The zeta potential for bacteria reflects the net surface charge, which is typically negative due to components like LPS, teichoic acids, and other anionic molecules on the cell wall. The less negative charge may have contributed to less interaction with the MNP, which is positively charged. Although the concentration factor was low for *E. coli* BAA-2471, it could still be detected by the biosensor.

The type 1 water, river water, and turkey rinse water samples were inoculated with ~10^5^ CFU of bacteria before magnetic capture. With a 40 mL sample volume, the estimated bacterial concentration in each sample was ~10^3^ CFU/mL. Previous study showed that 10^5^ CFU/mL was required for clinical detection of AMR in bacteria [71]. The final inoculated bacterial concentration in this study was 10^3^ CFU/mL, which was lower than in the previous study. After capture, the lowest DNA concentration recovered among the samples was 6 ng/μL. To ensure consistency in downstream testing, all samples were standardized to this minimum concentration. The type 1 water sample exhibited a higher CF compared to the river water and turkey rinse samples. One likely explanation is that MNPs in the river and turkey rinse samples bound not only to the target bacteria but also to naturally occurring microflora. However, since the MNP extracts were plated on selective media, these native microbes may not have grown, potentially leading to an underestimation of CF. Additionally, particulate matter in the complex matrices, such as dirt and sand in river water or food debris in the turkey rinse, may have interfered with MNP binding or hindered magnetic separation. Despite these differences in matrix composition and CF, the biosensor successfully detected DNA from bacteria at concentrations as low as ~10^3^ CFU/mL across all sample types. This is comparable to the limit of detection of other portable detection methods, such as the NG-test CARBA 5, where blood samples were inoculated with 10^4^ CFU/mL and incubated before detection [72]. For PCR, bacteria can be detected as low as 6–24 CFU/mL in a variety of food sample types [73]. However, this level of sensitivity often requires complex sample preparation, highly optimized procedures, and optimal laboratory conditions. This tradeoff between limit of detection and operational simplicity supports the biosensor’s potential as a complementary tool for preliminary screening in high-load environments, with PCR methods serving as a confirmatory assay when there are low bacterial loads. Antibiotic-resistant bacteria could exist in high concentrations in some environments, like wastewater treatment plants, and sulfamethoxazole-resistant bacteria were found in concentrations up to 10^7.2^ CFU/mL [74]. Another study found that in wastewater influent, the number of isolated bacteria ranged from 10^2^ to 10^5^ CFU/mL with a median of 2.5 × 10^4^ CFU/mL, but the percentages of samples with carbapenem-resistant bacteria ranged from 0 to 56.7% depending on species [75]. This indicates that, although the concentration of bacteria in water samples can be high, the level of resistant bacteria can be low. This biosensing method may be most useful in environments, like hospital wastewater, with a higher, potentially resistant bacterial load. Additional sensitivity testing may be required for application in high-load environments. Furthermore, strain-specific differences in surface structure can affect capture levels, which may limit the biosensor’s ability to detect diverse carbapenem-resistant bacteria in field applications.

To assess the biosensor’s global applicability, an in silico assessment was conducted using the Global Align website of the National Center for Biotechnology Information (NCBI, https://www.ncbi.nlm.nih.gov/ accessed 13 August 2025). These bacterial strains were isolated from various clinical samples, such as endotracheal aspirate, urine, blood, etc. Table 3 shows the alignment summary, including the bacteria containing the resistance gene, percent identity (alignment), coverage percentage, E-value, and bacterial isolation source. There were no gaps or misalignments observed in any of the following examples.

One limitation of this study is the lack of a multiplex experimental design. For future work, all three probes should be combined into a single tube to determine whether successful detection of the gene probes is possible. This would decrease the time and materials needed to run this biosensing method. Before multiplexing can be attempted, a cross-reactivity study should be performed for all three probes and target genes. Another limitation is that the matrix effect was not fully studied in terms of biosensor response and bacterial capture efficiency. For future work, experiments should include testing the response of the biosensor against non-inoculated samples to determine whether natural microflora influence response. Additional matrices, like food and clinical samples, should be tested to validate the biosensor response and matrix effect as well as clinical isolate testing to validate results in real samples.

## 5. Conclusions

The portable, inexpensive detection methods for carbapenem-resistant bacteria can improve surveillance efforts, especially in low-income countries. This GNP-based plasmonic biosensor assay detected the CR genes *bla*_NDM-1_, *bla*_OXA-1_, and *bla*_KPC-3_. To assess the portability and accessibility of the biosensor, the method included DNA extraction that did not require a kit, an RGB measurement cellphone application, and a portable thermal cycler. The biosensor provided visual differentiation between target bacteria containing resistance genes and non-target susceptible bacteria and controls magnetically extracted from water samples. The goal of magnetic extraction was to bypass lengthy sample preparation steps and provide a method for directly concentrating bacteria from samples. To digitally analyze the results, spectral data using the ratio of absorbance values at 520 nm and 620 nm (A_520_/A_620_) were used, in addition to a cellphone application measuring the RGB values. The novelty of this biosensor is in the parallel detection of CRGs using a simple boiling method for DNA extraction and portable thermocycler for a potential field-operable system. This system shows the potential for field and POC testing to aid in AMR surveillance initiatives and rapid diagnostics.

## Figures and Tables

**Table 2:** Average concentration factor capture efficiency obtained when comparing the plate counts from the sample solution before and after MNP addition and concentration in spiked type 1 water, river water, and turkey rinse.

Bacteria	Sample	Average Concentration Factor (*n* = 3)	Average Capture Efficiency % (*n* = 3)
*E. coli* BAA-2340	Type 1 Water	16.97	89.92
	River Water	1.75	49.47
	Turkey Rinse	1.15	47.03
*E. coli* BAA-2471	Type 1 Water	0.43	47.61
	River Water	0.10	36.29
	Turkey Rinse	0.26	44.57
*E. coli* C-3000	Type 1 Water	18.30	83.69
	River Water	2.70	74.61
	Turkey Rinse	1.72	61.57
*E. cloacae*	Type 1 Water	14.85	78.09
	River Water	7.36	85.28
	Turkey Rinse	1.14	52.09
*S. aureus*	Type 1 Water	17.18	85.26
	River Water	4.51	75.24
	Turkey Rinse	2.93	69.67

**Table 3:** Alignment study of *bla*_KPC_, *bla*_NDM-1_, and *bla*_OXA-1_ using the NCBI database.

Gene	Bacteria	Accession Number	Alignment %	Coverage %	E-Value	Isolation Source
*bla*_KPC_	*E. coli*	MF772496	100	100	$9\times 10^{-30}$	Urine culture
		NG_244524			$9\times 10^{-29}$	Respiratory sample
	*K. pneumoniae*	NG_049250			$9\times 10^{-30}$	Sputum sample
		NG_074720			$9\times 10^{-30}$	Pus sample
		KU216748				
	*P. aeruginosa*	PV103221			$9\times 10^{-30}$	Sputum sample
		PP784157			$9\times 10^{-30}$	Blood culture
		OM317762			$9\times 10^{-30}$	Drainage
		MK463614			$9\times 10^{-30}$	Water effluent
*bla*_NDM-1_	*E. coli*	MN701975	100	100	$1\times 10^{-22}$	Canine clinical samples
		KT749876			$2\times 10^{-24}$	Hospital effluent
		PV022994			$5\times 10^{-22}$	Rectal swab
	*K. pneumoniae*	KX218441			$2\times 10^{-24}$	Aspirate sample
		CP095585			$4\times 10^{-22}$	Blood culture
	*P. aeruginosa*	CP020703			$2\times 10^{-20}$	Sputum
		MF356396			$2\times 10^{-24}$	Urine sample
		CP194170			$2\times 10^{-20}$	Abscess sample
*bla*_OXA-1_	*E. coli*	CP056470	100	100	$1\times 10^{-20}$	Wastewater treatment effluent
		OL582571			$4\times 10^{-25}$	Bovine milk
		CP047662			$1\times 10^{-20}$	Fecal sample
	*K. pneumoniae*	PV075138			$2\times 10^{-24}$	Urine culture
		CP120891			$8\times 10^{-22}$	Blood sample
	*P. aeruginosa*	CP040126			$1\times 10^{-21}$	Fecal sample
		CP030913			$2\times 10^{-20}$	Sputum sample
		CP114762			$1\times 10^{-21}$	Blood sample

## Data Availability

The data presented in this study are available upon request from the corresponding author.
